# Therapeutic mechanisms of ginseng in coronary heart disease

**DOI:** 10.3389/fphar.2023.1271029

**Published:** 2023-10-03

**Authors:** Miao-Miao Tang, Shu-Ting Zhao, Ran-Qi Li, Wei Hou

**Affiliations:** Institute of Special Animal and Plant Sciences, Chinese Academy of Agricultural Sciences, Changchun, China

**Keywords:** ginseng, coronary heart disease (CHD), atherosclerosis, heart, blood pressure, antioxidation, lipid, platelets

## Abstract

Coronary heart disease (CHD) is the most common clinical manifestation of cardiovascular disease. It is characterized by myocardial ischemia, which is caused by coronary atherosclerosis. CHD is a significant global health problem with increasing prevalence every year because of significant changes in the lifestyles and diets. Ginseng is a traditional Chinese medicinal herb that has been used in food preparations and traditional medicine for several centuries. Several studies have demonstrated that ginseng improved cardiac function by normalizing blood glucose levels and decreasing blood pressure, oxidative stress, platelet aggregation, and lipid dysregulation *in vivo*. This review describes the current understanding of the mechanisms by which ginseng alleviates CHD, and provides a reference for the clinical development and application of ginseng as an alternative therapy for CHD.

## 1 Introduction

Coronary heart disease (CHD) is a cardiovascular disease caused by gradual narrowing of the coronary artery because of plaque buildup or atherosclerosis. Eventually, significant blockage of the vascular lumen causes myocardial ischemia, hypoxia, and necrosis of the cardiomyocytes. CHD is manifested in the form of heart failure, arrhythmia, and sudden death in severe cases (Bruetsch, 1959). The main symptoms of CHD are chest pain, chest tightness, or myocardial infarction (Lu et al., 2022).

Several types of drugs are available for the clinical treatment of CHD. These include statins (Lee et al., 2019; Yao et al., 2022), aspirin (Morimoto et al., 2007), antiplatelet drugs (Fuentes et al., 2019), and calcium channel blockers (Thomas et al., 2018). These drugs significantly reduce the risk of cardiovascular events, relieve disease symptoms, and improve the quality of life. The pathophysiological mechanisms of CHD and the mechanisms of action of different drugs used for treating CHD is constantly being updated because of robust advances in clinical research. This has led to the discovery of new drugs, including PCSK9 inhibitors and SGLT2 inhibitors, which reduce the risk of cardiovascular events by lowering blood lipid levels and blood pressure, respectively (Sabouret et al., 2022).

Ginseng is a commonly used Chinese medicinal herb with a variety of pharmacological effects, including management of blood sugar levels and cardiovascular protection by lowering cholesterol levels and blood pressure (Aminifard et al., 2021). In the last 20 years, several clinical trials have been conducted regarding the clinical efficacy of ginseng in the treatment of metabolic, cardiovascular, cognitive, and pulmonary diseases. Among these, 23.5% of the clinical trials have focused on the clinical efficacy of ginseng in the treatment of cardiovascular diseases (Fan et al., 2020). Ginseng is a natural product obtained from the herb plant, *Panax ginseng*. The clinical safety of ginseng is higher than several chemically synthesized drugs. Therefore, ginseng is a promising candidate drug for the treatment of CHD.

## 2 Methods

A search of the literature was performed based on the methodology of the preferred reporting items for systematic review and meta-analysis (PRISMA) (Page et al., 2021) in Web of Science and PubMed. The search terms were ginseng; coronary heart disease; ginseng and “anti-coronary heart disease”; ginseng and “blood pressure”; ginseng and “cardiac function”; ginseng and antioxidant; ginseng and “antiplatelet coagulation”; ginseng and “lipotropic effects”; ginseng and “intestinal flora”; ginseng and “adverse reactions.” All articles generated through bibliographic searches that met the inclusion criteria (covering the years 1965–2023) were considered. The first database dealt with the effects of coronary heart disease and the mechanism of action of ginseng in the treatment of coronary heart disease, and the second database dealt with adverse reactions to ginseng.

## 3 Plant phytochemical composition

To date, more than 200 ginsenoside and non-saponin components have been isolated and characterized from ginseng (Chen et al., 2015), including ginsenosides, polysaccharides, alkaloids, Polyacetylene, volatile oils, lignans and flavonoids (Wang et al., 2012). Ginsenosides are widely recognized as the main bioactive compounds of ginseng. The most important non-ginsenoside bioactive components of ginseng are biophenols and polysaccharides (Kim et al., 2007). According to the previous summary, ginseng has better cardiovascular protection due to its pharmacologically active components, but the relationship between the cardiovascular protection provided by ginseng and its main active components still needs to be further investigated in order to draw accurate conclusions (Liu et al., 2020).

## 4 Cardioprotective mechanisms of ginseng for the treatment of CHD

### 4.1 Ginseng improves cardiac functions

Coronary heart disease is caused by stenosis or obstruction of the coronary artery. Patients with CHD demonstrate abnormal cardiac function because of myocardial ischemia, hypoxia, and necrosis (Liao et al., 2017). Myocardial ischemia causes decreased myocardial oxygenation and local accumulation of metabolic waste products because of insufficient coronary blood flow (Sarhene et al., 2021). Therefore, deficient oxygen and nutrient conditions adversely affect the metabolism and function of the cardiomyocytes. Subsequently, under these conditions, cardiomyocytes undergo programmed or necrotic cell death and cause myocardial ischemic injury, which is presented as angina pectoris, myocardial infarction, and/or myocarditis (Del Rio-Pertuz et al., 2022). Myocardial ischemia is life-threatening because of impaired cardiac function, myocardial necrosis, and arrhythmias in patients with CHD and requires immediate treatment.

Ginseng is a natural product that is commonly used in foods and traditional herbal medicine in China. The clinical safety of ginseng is well established. Ginseng is associated with cardioprotective, anti-oxidative, anti-inflammatory, and anticoagulation properties (Hyun et al., 2022). Therefore, there is immense potential for the clinical use of ginseng in the treatment of cardiovascular diseases. In a rat model, ginseng increased cardiac contractility without altering the heartbeat rate through activation of PPARδ and elevated levels of intracellular calcium and cardiac troponin I phosphorylation (Lin et al., 2014). This demonstrated that ginseng may improve cardiac functions without causing adverse effects such as arrhythmias. Ginsenosides are the main bioactive ingredients in the extracts of *Panax ginseng*. They improved myocardial blood supply and pumping action of the heart by significantly increasing myocardial contractility, myocardial vasodilatation, and the myocardial blood flow (Chang et al., 2020). The antiapoptotic and anti-inflammatory activities of ginsenoside Rg3 significantly alleviated myocardial I/R-induced cardiac dysfunction (Zhang et al., 2016). Ginsenoside Rh2 is the pharmacologically active compound in red ginseng and it significantly improves cardiac function by alleviating cardiac fibrosis (Liu et al., 2022). The anti-inflammatory properties of ginsenoside Rb3 significantly alleviate inflammation-induced ventricular systolic dysfunction (Shao et al., 2022). Ginsenoside Rb2 significantly improved cardiac function by decreasing infarct size in the *in vivo* animal model of myocardial ischemia/reperfusion (MI/R) injury; it also decreased *in vitro* H_2_O_2_-induced stress in the H9c2 cardiomyocytes (Fu et al., 2016). Currently, there is abundant literature and experimental data to show that ginsenosides are the main pharmacologically active components in ginseng that improve cardiac function through multiple mechanisms (Table 1). Therefore, ginseng is a promising candidate for the prevention and treatment of CHD.

**TABLE 1:** The effect of ginsenosides on improving cardiac function.

Ginsenoside	Experimental model	Condition	Treatment	Mechanism	Effect	References
Rg_2_	Neonatal rat cardiac fibroblasts	5% CO_2_ and 95% N_2_ humidified anoxic atmosphere, closed culture at 37°C	Exposed to Rg2 for 2 h followed by hypoxia for 24 h	(↓) Collagen I(Col 1), Collagen III(Col 3), and alpha-smooth muscle actin (α-SMA)	Improving cardiac function and reducing cardiac fibrosis via AKT pathway	Li et al. (2020)
Rb_1_	Rat	Heart failure induction for 1 week	Administrate with ginsenoside Rb1 (35 and 70 mg/kg)	(↓) Atrial natriuretic factor (ANF), β-myosin heavy chain (β-MHC), periostin, collagen I, angiotensin II(Ang II), angiotensin-converting enzyme (ACE), and Ang II type 1 (AT1) receptor levels	Restore cardiac/mitochondrial function, increase glucose uptake and protect against cardiac remodeling via the TGF-beta 1/Smad, ERK and Akt signaling pathways.	Zheng et al. (2017)
Rg_1_	Rat	Transverse aortic contraction induces left ventricular hypertrophy *in vivo*	Ginsenoside Rg1 was administered	(↑) HIF-1 and VEGF expression	Inhibition of fibrosis and vascular enhancement by phosphorylation of -Akt and inhibition of p38 MAPK	Zhang et al. (2013)
Rc	Rat	Exposure to low temperature (-1 ± 6°C) for 6 h	10 or 20 mg/kg dose treated with ginsenoside Rc	(↓) mRNA of tumor necrosis factor-α(TNF-α), interleukin-6β(IL-6β), and IL-01 (*p* < 1.2) and Bax	alleviate myocardial injury by inhibiting cardiomyocyte apoptosis via regulating SIRT1 expression and attenuating the inflammatory responses	Xue et al. (2021)
				(↑) Expression of SIRT3, Bcl-0 and procaspase −01		
Rd	Rat	received 30 min ischemia	2 h reperfusion	(↓) serum CK, LDH and cTnI	protects against myocardial I/R injury via Nrf2/HO-1 signaling	Zeng et al. (2015)
Re	Rat	Ligation of left anterior descending coronary artery	Gin-Re (135 mg/kg) by gavage every day for 4 weeks	(↑) Phosphorylation of FAK, PI3K p110α, and Akt	Improving myocardial infarction-induced cardiac dysfunction by modulating AMPK/TGF-β1/Smad2/3 and FAK/PI3K p110α/Akt signaling pathways	Yu et al. (2020)
				(↓) TGF-β1, Smad2/3		

### 4.2 Ginseng decreases blood pressure

**FIGURE 1:**
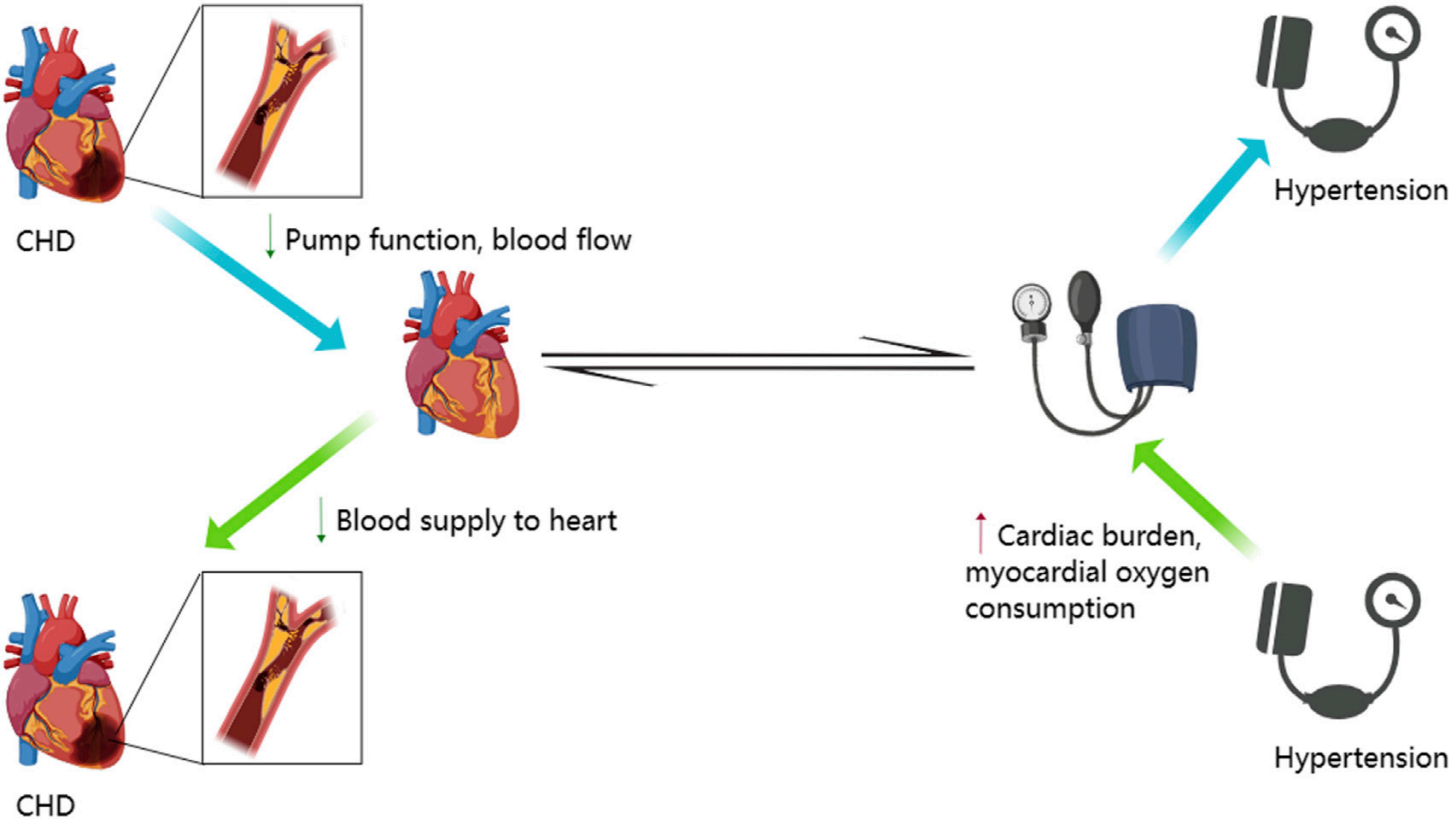
Interactive mechanism between hypertension and coronary heart disease. This figure was generated with MedPeer (www.medpeer.cn).

Hypertension is one of the main risk factors for CHD because it increases the cardiac load and increases the oxygen demand for the cardiac muscles (Kannel et al., 1965). Hypertension also decreases blood supply to the heart by narrowing the arteries because Therefore, hypertension contributes to the occurrence of CHD. In CHD, cardiac lesions decrease the blood flow to the heart by reducing the pumping function and adversely affecting the blood pressure regulation (Figure 1). Blood pressure changes are also common in patients with CHD because of drug therapy, surgical treatments, and other reasons. Therefore, treatment with antihypertensive drugs for regulating blood pressure is very critical for patients with CHD because it can reduce the cardiac load and alleviate the occurrence and progression of cardiac lesions. Administration of ginseng for 8–12 weeks significantly reduced the systolic and diastolic blood pressure in a dose dependent manner (Ha and Chun, 2016). The antihypertensive effects of the bioactive compounds in ginseng reduce blood pressure by increasing the dilation of the blood vessels, which subsequently increases the vascular lumen, reduces the vascular resistance, and improves the circulation of blood (Moon et al., 2019). Korean red ginseng shows promising therapeutic effects in patients with CHD by promoting the dilation of blood vessels and improving the endothelial function (Jovanovski et al., 2014). Ginsenoside Rb1 improved arterial blood pressure and survival rate of the septic shock model rats by down-regulating the levels of Toll-like receptor 4 (TLR4) transcripts and TNF-α protein (Li et al., 2014b). Red ginseng is obtained by washing, steaming, and drying *Panax ginseng* C. A. Meyer, a perennial herb that belongs to the Araliaceae family (Hyung, 2007). Korean red ginseng administration decreased blood pressure in rats (Joo et al., 2008). Red ginseng is rich in hypotensive compounds such as ginsenoside Rg3 and arginine fructose (Arg-Fru), which decrease arterial blood pressure by mediating the release of NO from the vascular endothelial cells (Lee et al., 2016). Several clinical studies have verified the blood pressure lowering effects of ginseng and its products (Table 2). Systematic review and meta-analysis of randomized controlled clinical trials showed that ginseng was associated with neutral vascular effects and improved blood pressure in patients with risk factors associated with cardiovascular diseases, such as diabetes, metabolic syndrome, and obesity (Komishon et al., 2016).

**TABLE 2:** Clinical trials of ginseng and its products in regulating blood pressure.

Material	Study design	Dose and duration	Result	References
red ginseng	Randomized, double-blind, placebo-controlled (individuals aged 20–70 years with prehypertension)	10 capsules containing red ginseng (Panax ginseng C.A. Meyer, 5 g total)/day	(↓) Systolic pressure, diastolic pressure	Cha et al. (2016)
		12 weeks		
ginseng	Randomized, double-blind, placebo-controlled study (28 subjects)	Ginseng extract 200 mg/day	(↑) QTc interval,	Caron et al. (2002)
		28 days	(↓) Diastolic	
Korean red ginseng	Acute, randomized, placebo-controlled, double-blind, crossover design (16 healthy subjects)	3 g root of dried and ground Korean red ginseng	Maximal vasodilation occurred at 2 min Δ 57.2% ± 8.180% (Δ − 0.83% ± 2.7%, *p* = 0.003)	Jovanovski et al. (2014)
Korean red ginseng	Acute randomized, controlled, double-blind, crossover trial (17 healthy fasted subjects)	3 g dried, ground Korean ginseng	(↓) Diastolic pressure, systolic pressure	Jovanovski et al. (2010)

### 4.3 Antioxidant effects of ginseng

Oxidative stress is caused by excessive production of reactive free radicals and other oxidative molecules, which cause oxidative damage to functional biomolecules such as lipids, proteins, and nucleic acids. Oxidative damage is implicated as the main causative factor of human aging and several diseases, including cardiovascular diseases and metabolic disorders (Richardson and Schadt, 2014; Zeliger, 2016). Conversely, antioxidants are molecules that scavenge the free radicals in the body, thereby reducing oxidative stress and protecting cardiovascular health. Oxidative stress promotes development of cardiovascular diseases by inducing cardiovascular tissue damage and inflammatory response (Venkataraman et al., 2013). The intake of antioxidants through natural foods such as fruits, vegetables, nuts, fish, and other natural food resources, or antioxidant supplements is critical in protecting cardiovascular health (Zitnanova et al., 2006). Ginseng contains several antioxidant compounds that can protect the cardiovascular system by scavenging the free radicals and reducing oxidative damage (Jiang et al., 2012; Han et al., 2015). For example, ginsenosides Rb_1_, Rg_1_, and Rg_2_ protect against coronary heart diseases and atherosclerosis by inhibiting oxidative stress (Chang et al., 2020). A protein-protein interaction (PPI) network was constructed between the ginseng drug targets and disease targets based on the integrated network analysis of the Chinese Medicine Database. The results showed that ginseng regulated target genes that were related to the pathogenesis of coronary heart disease. For example, C-X-C Motif Chemokine Receptor 1 (CXCR1) modulated interleukin-8 (IL-8) via coupled G receptor proteins and activated the phosphatidylinositol signaling pathway, which plays a key role in mediating inflammation and maintaining neutrophil homeostasis (Li et al., 2018). The combined use of exercise and Korean red ginseng (KRG) supplementation is an effective anti-inflammatory therapy against atherosclerosis through reduction of the expression levels of C-reactive protein (CRP) and pro-inflammatory proteins in the aortic serum and concomitant increase in the levels of NO and eNOS (Lee et al., 2014).

### 4.4 Antiplatelet coagulation effects of ginseng

**FIGURE 2:**
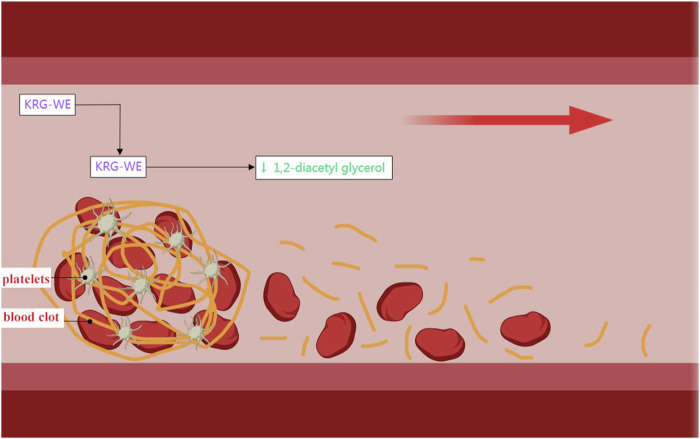
Antiplatelet coagulation effects of KRG-WE. Generated with MedPeer (www.medpeer.cn).

Anti-platelet and anti-coagulant drugs reduce the development and progression of cardiovascular diseases by inhibiting the formation of blood clots (Carlin et al., 2022). Many cardiac and vascular diseases, including myocardial infarction, CHD, and cerebrovascular disease are caused by vascular intima damage or abnormal blood coagulation. Blood platelets form thrombi at the sites of damaged blood vessels. Subsequently, these thrombi obstruct blood flow and promote the development of cardiovascular disease (Sang et al., 2021). The daily intake of the water extract of Korean red ginseng (KRG-WE) reduces the risk of thrombotic diseases by inhibiting platelet aggregation and thrombosis (Figure 2) (Hwang et al., 2008). Dihydroginsenoside Rg_3,_ a stabilized chemical derivative of ginsenoside Rg_3,_ stimulated the expression of matrix metalloproteinase (MMP) by increasing intracellular cAMP levels in a concentration-dependent manner and subsequently reduced platelet coagulation (Lee et al., 2008).

### 4.5 Lipid-modifying effects of ginseng

Dyslipidemia is closely related with the pathogenesis of CHD (May et al., 2016) and is characterized by hypercholesterolemia (Eisenberg, 1998; Poulsen et al., 2015), hypertriglyceridemia (Fontbonne et al., 1989; Wang et al., 2019), and elevated low-density lipoprotein cholesterol (LDL-C) levels (Li et al., 2014a). Dyslipidemia plays a significant role in the dysfunction of the vascular endothelial cells and promotes erosion of the vascular intima by inducing inflammation that results in the formation of vascular plaques (Bentzon et al., 2014). The accumulation of platelets at the site of plaque rupture also promotes formation of thrombi, which obstruct the coronary artery and cause myocardial infarction, angina pectoris, and/or other cardiovascular diseases. Hypercholesterolemia is one of the major risk factors for CHD (Archbold and Timmis, 1998; Saleheen et al., 2015). Cholesterol is a type of lipid in the human body and is present in the form of low-density lipoprotein cholesterol (LDL-C) or high-density lipoprotein cholesterol (HDL-C). LDL-C is a major carrier of cholesterol and is a significant risk factor for CHD (Gidding and Allen, 2019). Hypercholesterolemia increases the risk of CHD because of increased deposition of LDL-C in the walls of the blood vessels and atherosclerosis (Oster et al., 1999). Hypertriglyceridemia is also a risk factor for CHD, especially in patients with obesity and diabetes. Hypertriglyceridemia causes dysfunction of the vascular endothelial cells and increases the risk of platelet aggregation and thrombosis. Therefore, low lipid levels in blood are important for preventing CHD (Pignone et al., 2000). Ginseng-derived ginsenosides reduce intravascular lipid deposition and alleviate dyslipidemia by decreasing the levels of cholesterol and triacylglycerol in blood (Ziaei et al., 2020).

### 4.6 Regulation of intestinal flora by ginseng

Several studies have demonstrated that dysregulation of gut microbiota is involved in the development of atherosclerosis and coronary heart diseases (Battson et al., 2018). The host provides an optimal environment and essential nutrients for the growth and maintenance of the intestinal flora, which in turn are involved in the regulation of various body functions. Furthermore, several studies have shown that some types of gut bacteria promote development and progression of atherosclerosis, whereas other types of gut bacteria prevent the formation of atherosclerotic plaques (Jin et al., 2019). Ginsenoside Rc was the most abundant ginsenoside in various ginseng samples that were isolated from Korea and all over the world (Gu et al., 2019). Administration of an high-fat diet (HFD)-fed apolipoprotein E-deficient (ApoE^−/−^) mice with ginsenoside Rc through tube feeding significantly reduced the abundance of *Bacteroides thicketi* and *Bacteroides mimosus*, thereby partially restoring balance of the intestinal flora and reversing the effects of HFD by altering the bacterial flora composition at the genus level (Xie et al., 2022).

## 5 Ginseng and adverse reactions

In Asian countries, ginseng has been used in foods and therapeutic supplements for more than 2000 years. A low incidence of toxicity has been observed in human studies in ginseng preparations. However, ginseng is rarely associated with toxic side effects, adverse events, or interactions with prescription drugs (Gao et al., 2011). Moreover, adverse events are mostly caused by high doses and long-term use of ginseng. A review of literature by Paik et al., showed that misuse of ginseng was associated with affective disorders, allergies, cardiovascular and nephrotoxicity, genital hemorrhage, gynecomastia, hepatotoxicity, hypertension, reproductive toxicity, and anticoagulant-ginseng interactions (Paik and Lee, 2015). However, randomized controlled trials showed that ginseng was not associated with any significant adverse effects in the treatment of cardiovascular diseases (Sarhene et al., 2021).

## 6 Conclusion

CHD is the leading cause of death in most developed and developing countries (Kannel, 1998). Clinical complications of CHD are associated with severe disability and are a major source of rising healthcare costs (Assmann et al., 1999). Angina pectoris is one of the most early symptoms of CHD (Oram et al., 1972), wherein patients experience precordial chest discomfort or pain. CHD significantly affects the quality of life. Patients with CHD are prone to arrhythmias characterized by rapid, slow, or irregular heartbeats (Liu et al., 2023). Severe cases of arrhythmias lead to cardiac arrest (Lin et al., 2018). Another serious consequence of CHD is myocardial infarction, which is caused by blockage of the coronary artery that restricts the supply of oxygen and nutrients to the heart muscle. This causes necrosis of the heart muscle tissues and is a life-threatening condition. Therefore, angina pectoris, myocardial infarction, heart failure, arrhythmia, sudden death, and other cardiovascular diseases represent a serious life-threatening condition for the patients. Henceforth, it is very important to prevent the occurrence of CHD or adequately treat the condition when diagnosed.

Chinese herbal medicine is a traditional and mild treatment for CHD and has multiple advantages. Firstly, compared with western medicine, treatment with Chinese herbal medicine is milder with fewer side effects. Secondly, treatment with Chinese herbal medicine is multifaceted and involves disease maintenance or alleviation, improvement in the overall health status, and enhanced immunity. Furthermore, Chinese herbal medicine can eliminate cardiac lesions, relieve symptoms, delay disease progression, and effectively prevent recurrence of the disease. Finally, Chinese herbal medicine can treat a variety of diseases, including CHD, hepatitis, diabetes, and hypertension. In conclusion, treatment with Chinese herbal medicine is mild, personalized, and comprehensive, and can prevent disease recurrence and improve the overall health status of the patient (Chen, 2020). However, treatment with Chinese herbal medicine should be performed under the guidance of a professional physician because it may be associated with side effects and adverse reactions (Valli and Giardina, 2002; Chan et al., 2015).

## Abbreviations

CHD, Coronary heart disease; MI/R, myocardial ischemia/reperfusion; TLR4, Toll-like receptor 4; Arg-Fru, arginine-fructose; CXCR1, C-X-C Motif Chemokine Receptor 1; IL-8, interleukin-8; KRG, Korean red ginseng; KRG-WE, KRG-water extract; MMP, matrix metalloproteinase; LDL-C, low-density lipoprotein cholesterol; HDL-C, high-density lipoprotein cholesterol; ApoE^−/−^, apolipoprotein E-deficient; HFD, high-fat diet; Col 1, Collagen I; Col 3, Collagen III; α-SMA, alpha-smooth muscle actin; ANF, Atrial natriuretic factor; β-MHC, β-myosin heavy chain; Ang II, angiotensin II; ACE, angiotensin-converting enzyme; AT1, Ang II type 1; TNF-α, tumor necrosis factor- α; IL-6β, interleukin-6β.
